# Domain-Specificity of Educational and Learning Capital: A Study With Musical Talents

**DOI:** 10.3389/fpsyg.2020.561974

**Published:** 2020-09-25

**Authors:** Marold Reutlinger, Wolfgang Pfeiffer, Heidrun Stoeger, Wilma Vialle, Albert Ziegler

**Affiliations:** ^1^Faculty of Humanities, Social Sciences, and Theology, University of Erlangen-Nuremberg, Erlangen, Germany; ^2^School Research, School Development, and Evaluation, University of Regensburg, Regensburg, Germany; ^3^Faculty of Social Science, University of Wollongong, Wollongong, NSW, Australia

**Keywords:** music, talent development, educational capital, learning capital, domain specificity

## Abstract

The Education and Learning Capital Approach (ELCA) has been widely used to investigate talent development. A research gap is the implicit consideration of the domain specificity of educational and learning capital. In an empirical study with 365 school students we investigated the domain specificity of the approach for the domains of school learning and learning to play a musical instrument. At the beginning of the school year, students filled out a version of the Questionnaire for Educational and Learning Capital (QELC) for both domains and also responded to other domain-related measures (self-efficacy, grades). Six weeks later, students filled out a learning diary for 1 week in which they reported their activities on an hourly basis and responded to questions concerning these activities. Based on the Sociotope Approach this procedure helped to identify times in which students actually practiced their musical instrument, times that students could potentially practice their musical instrument (objective action space), and times that students would be expected to practice their musical instrument (normative action space). Three hypotheses were tested and could be supported. First, the availability of educational and learning capital for school learning and learning an instrument differed. Second, a confirmatory factor analysis supported the factorial validity of the domain-specific capital measurements. Third, domain-congruent correlations were mostly higher than domain-incongruent correlations, i.e., the availability of educational and learning capital for school learning correlated more closely with variables related to school learning than with variables related to learning a musical instrument. Similarly, the availability of the capitals for learning a musical instrument correlated more closely with variables related to learning a musical instrument.

## Introduction

Two key insights on talent development are that people can differ substantially in both the speed of skill acquisition and the level of performance ultimately achieved (VanLehn, 1996; Ericsson et al., 2006; Shavinina, 2009; Attri, 2019). Since its beginnings, there has been a strong tendency in talent and giftedness research to explain these phenomena with domain-general concepts such as talents, gifts, and IQ (Galton, 1883; Terman, 1925, 1954; Hollingworth, 1942; Howe et al., 1998). Even today, the echo of these beginnings is still noticeable, especially in practice. For example, in gifted identification, the general intelligence quotient – next to general performance indicators such as GPA – is still the most important indicator (Ziegler et al., 2018).

For decades, however, many studies have shown that human learning and action cannot be fully understood if the unit of analysis is the decontextualized individual (Leont’ev, 1978; Vygotsky, 1978; Scribner, 1984; Suchman, 1987; Lave, 1988; Norman, 1988; Newman et al., 1989; Salomon, 1993). The idea that not only talents and gifts are important, but also what the individual applies these talents and gifts to was taken up very quickly. Numerous new concepts were proposed. Gardner’s conception of multiple intelligences exerted a great influence. He postulated seven and later even more domain-specific intelligences (Gardner, 1983, 1986; Gardner and Moran, 2006). Other researchers like Tannenbaum (1986), Gagné (1993), and Heller et al. (2005) or Subotnik et al. (2011) postulated not only specific abilities, but rather specified and included domains in their models of giftedness and talent development. For example, Heller et al. (2005) mentioned mathematics, natural sciences, technology, computer science, art, languages, sports, and social relationships as domains.

In addition to naming domains, the narrow focus on domain-general personality factors was broadened by researchers. With regard to the person and the environment (as well as their interaction), a more holistic perspective was adopted (for an overview refer to Stoeger et al., 2017a). Numerous researchers suggested non-intellectual personality traits that should be incorporated into conceptions of giftedness. Examples include bodily-kinesthetic and interpersonal abilities (Gardner, 1983), creativity and task commitment (Renzulli, 1986), secure self-concept and persistence (Tannenbaum, 1986). In a similar vein, some researchers have explicitly included environmental factors in their conception of giftedness. This usually took the form of social units such as family or peers or social settings such as school (Mönks, 1992). It was assumed that these personality traits and environmental factors then acted as catalysts (e.g., Gagné, 1993) or moderators (e.g., Heller et al., 2005), which are crucial in transforming talents and gifts into high performance levels in the domains.

The main outcome of these theoretical developments at the end of the last century was that three standards were set that are still widely in place today: The holistic view of the person, the incorporation of the environment, and the importance of person-environment-interactions (Pfeiffer, 2018; Pfeiffer et al., 2018).

A number of new conceptions of talent development have been proposed that respect these three standards, focusing particularly on the interaction between the individual and the environment (Ziegler and Stoeger, 2017a; Ziegler et al., 2017b; Lo et al., 2019; Mudrak et al., 2019; Crawford et al., 2020; Dai, in press). These models are in line with Csikszentmihalyi’s dictum (1998), according to which creative eminence is no longer only localized in the person, but in the system of the person and her environment. Person and environment are in this sense no longer separate entities, but interacting components of systems. These systems contain as a central component, the particular domain in which eminence is achieved.

Still, the central question is how an individual within a certain environment can achieve extraordinary performance levels in a particular domain. One answer given by many researchers is the availability of resources (Chandler and Ziegler, 2017; Phillipson et al., 2017; Stoeger et al., 2017b; Vialle, 2017; Lafferty et al., 2020; Paz-Baruch, 2020). However, the only fully elaborated resource-oriented approach to talent development to date is the Educational and Learning Capital Approach (ELCA) proposed by Ziegler and colleagues (Ziegler and Baker, 2013; Vladut et al., 2015; Ziegler et al., 2017a; Ziegler and Stoeger, 2019). Strangely enough, although there are various studies that investigate the role of these resources for talent development in different domains (e.g., Debatin et al., 2015; Stoeger et al., 2017b; Ziegler et al., 2019), the question of the domain specificity of these resources has not been explicitly addressed so far. Filling this gap is the objective of our study.

### Learning Resources in Talent Development

The Education and Learning Capital Approach starts from the observation that many aspects of talent development and eminence that have been scientifically studied do not occur randomly, but in clusters. The most comprehensive level of analysis where such clusters have been found so far is “Golden Ages” (Pfleiderer, 1877)^1^. There are two well-known examples for eminence clusters in the domain of music. The first cluster includes Albinoni, Haendel, Vivaldi, Caldero, Cimarosa, Galuppi, Hasse, Jommelli, Lotti, the Marcello brothers, Parpora, Quantz, the two Scarlatti brothers, and Tartini. They all were active in 18th Century Venice within a 50-year period. A second, contemporary example of an eminence cluster in the domain of music includes well-known musicians and bands from London during the third quarter of the 20th century, such as David Bowie, Cat Stevens, The Byrds, Kinks, Motorhead, Nirvana, The Police, The Who, Rolling Stones, Sex Pistols, George Michael, Phil Collins, Peter Frampton, Elton John and many others.

Clusters of eminence in music—and also in other domains—can not only be identified within certain time periods but also at many other levels of analysis, including:

• Places, i.e., famous musicians are not distributed geographically at random, but group in selected places such as thriving cities (Schich et al., 2014).
• Institutions, i.e., some institutions constantly and frequently produce eminent musicians such as the Meadowmount School of Music in upstate New York, which counts Yo-Yo Ma, Pinchas Zuckerman, Joshua Bell, and Itzhak Perlman among its students (Coyle, 2009).
• Mentors, i.e., some people mentor an amazing number of outstanding artists. One example is Don Grierson, who has worked with the Beatles, Kim Carnes, Kate Bush, Cliff Richard, Joe Cocker, and Tina Turner. He is also considered the discoverer of Celine Dion and other major talents (Grierson and Kimpel, 2009).
• Masterpieces, i.e., some musicians are responsible for a disproportionate number of the most famous pieces of music. For example, The Beatles alone produced 23 songs from Rolling Stone’s 500 greatest songs of all time (Rolling Stone, 2008). Furthermore, John Lennon, Paul McCartney and George Harrison are also listed as solo artists.

These examples illustrate that there are not only differences in talent and giftedness between individuals, in terms of how likely they are to achieve eminence, as was originally assumed in giftedness research. Obviously, there are also differences between clusters, such as certain times, places or institutions that are more likely to favor the development of eminence. But what distinguishes these clusters? The answer from resource-oriented talent researchers would be that learning resources are the distinguishing aspect. Anecdotal data both in biographical and historio-metric analyses (e.g., Ochse, 1990; Simonton, 1994, 1999, 2019; Csikszentmihalyi, 1996) as well as numerous studies within expertise and talent research (Ericsson et al., 2018; Shavinina, 2009; Paik et al., 2019; Subotnik et al., 2019) support this claim for a wide range of learning resources such as mentors, family background, and motivation. ELCA is an attempt to compile and theoretically integrate the multitude of information that learning resources play for talent development.

The Education and Learning Capital Approach was developed within the framework of the Actiotope Model of Giftedness (Ziegler, 2005). According to this model, the basic unit of analysis of talent development is the actiotope, i.e., the individual and the segment of the material, social and informational environment with which she interacts (Ziegler et al., 2013). In such an individual lifeworld or “actiotope,” factors that enable successful talent development are understood as resources. They are therefore means to an end, the end being talent development (Ziegler et al., 2017a).

In ELCA, two types of resources are distinguished (Ziegler and Baker, 2013). Exogenous resources, which are located in the enacted environment, are called educational capital. Endogenous resources that are localized in the individual are called learning capital. ELCA postulates five forms of educational capital (economic, cultural, social, infrastructural, and didactic educational capital) and five forms of learning capital (organismic, telic, actional, episodic, and attentional learning capital). Definitions for each capital can be found in Table 1. Within the forms of educational capital and learning capital, economic educational capital and organismic learning capital play a special role. They are called proto-capitals (Ziegler et al., 2017a), because they must first be transformed into other capitals to promote talent development. For example, money does not directly promote talent development. However, it can be used to pay, for example, private teachers for music lessons, who then represent social educational capital. Music teachers, in turn, provide cultural and didactic educational capital and provide also access to and optimal use of infrastructural resources.

**TABLE 1:** Definitions of the various types of educational and learning capital according to Ziegler and Baker (2013).

Exogenous Resources		Endogenous Resources	
Type	Definition	Type	Definition
Economic educational capital	Economic educational capital denotes every kind of wealth, possession, money, or valuable that can be invested in the initiation and maintenance of educational and learning processes. (p. 27)	Organismic learning capital	Organismic learning capital denotes the physiological and constitutional resources of a person. (p. 29)
Cultural educational capital	Cultural educational capital denotes value systems, thinking patterns, models, and the like that can facilitate—or hinder—the attainment of learning and educational goals. (p. 27)	Telic learning capital	Telic learning capital denotes the totality of a person’s anticipated goal states that offer possibilities for satisfying her needs. (p. 30)
Social educational capital	Social educational capital denotes all persons and social institutions that can directly or indirectly contribute to the success of learning and educational processes. (p. 28)	Actional learning capital	Actional learning capital denotes the action repertoire of a person; as such, it describes the totality of actions a person is capable of performing. (p. 30)
Infrastructural educational capital	Infrastructural educational capital denotes materially implemented possibilities for action that allow learning and education to take place. (p. 28)	Episodic learning capital	Episodic learning capital denotes the simultaneous goal-relevant and situation-relevant action patterns that are accessible to a person. (p. 31)
Didactic educational capital	Didactic educational capital denotes the assembled knowhow involved in the design and improvement of educational and learning processes. (p. 29)	Attentional learning capital	Attentional learning capital denotes the quantitative and qualitative attentional resources that a person can apply to learning. (p. 31)

The role of educational and learning capital for talent development has been corroborated in numerous research studies. For example, in the domain of academic learning it has been shown that average students, high-performing students and underachievers differ in their resource profiles. Better talent development was associated with a more positive resource profile (Harder et al., 2015; Leana-Taşcılar, 2015b; Paz-Baruch, 2015, 2020; Vladut et al., 2015; Stoeger et al., 2017b; Ziegler and Stoeger, 2017b; Veas et al., 2018). Similar findings have been reported in other domains, including music, sports, and vocational success (Ziegler et al., 2014, 2019). In each of these studies, a domain-specific adaptation of the research material was used. It was implicitly assumed that a characteristic set of specific learning resources must be available for successful talent development in each domain. This means, for example, that talent development in music, football, painting, and mathematics require different learning resources. In fact, however, the need for such domain-specific adaptations and the availability of domain specific resources for talent development has not yet been explicitly demonstrated for educational and learning capital. This research deficit will be addressed in our study.

## Current Research

During talent development, endogenous and exogenous learning resources co-evolve in a process of circular causality (Bateson, 1972; Ziegler and Stoeger, 2017a). The processing of exogenous resources changes the endogenous resources, while the endogenous resources couple back through actions. Resources are thus connected in a characteristic and distinctive way and their interactions are coordinated. Their functionality is measured by how they influence talent development in a domain (Ziegler et al., 2017a).

If one extends this perspective to learning resources for two different domains, mutual influences of learning resources of the two domains must be considered. A learning resource of one domain A can either have a positive (+) or negative (−) effect on talent development in another domain or no effect on talent development in that domain at all (±). In this reciprocal process, the learning resource itself can remain positive (+), negative (-) or unchanged (±) in its effects on talent development in the original domain. In principle, learning resources from two domains can thus have six different relationships to each other: neutral (± ±), synergetic (+ +), destructive (− +), catalytic (± +), explosive (+), allostatic (±) (for exact definitions of these relations, see Ziegler and Stoeger, 2019).

However, the mutual effects of learning resources from different domains on talent development will always be a mix. One example is learning for school and learning to play a musical instrument. The relation can be destructive, i.e., both domains hinder each other with regard to the resource of time. Time used for learning for school may be lacking for practicing the musical instrument and vice versa. On the other hand, learning a musical instrument can be advantageous for a good grade in the school subject music and vice versa, good music lessons at school can be supportive for learning a musical instrument. In this case, the relation would be synergetic. As these examples show, it can be assumed that although each domain has characteristic resource profiles, these profiles themselves may not be completely independent of each other.

In our study, we investigate the domain specificity of educational and learning capital as well as relations between educational and learning capital in different domains. In designing the empirical study, we were guided by several research strategic considerations. First, we assumed that each person has resources that are differently functional for learning in different domains. Thus, it can be the case that the very same resource would be a learning resource with respect to domain A, but not with respect to domain B. To empirically demonstrate such an effect, a within-subject-design has to be chosen, in which a person’s learning is examined in two different domains.

We tried to find two domains, which, although needing different learning resources, are not too different. From a research strategy perspective this is important because if the need to take the domain specificity of learning resources into account can already be shown in rather closely related domains, then this also applies a fortiori to domains that are further apart.

We opted for the two domains of school learning and learning a musical instrument. The choice of these two domains was also based on the availability of measuring instruments. In a study like ours educational and learning capital for different domains should be assessed by comparable measuring instruments. A school-based version of the Questionnaire for Educational and Learning Capital (QELC; Vladut et al., 2013; Paz-Baruch, 2015; Arilena and Leana-Tacilar, 2016) and a parallel version formulated for the domain of learning a musical instrument were already available (Ziegler et al., 2014).

In summary, our research’s strategic considerations led to the decision that students who play a musical instrument should work on both the school and music versions of QELC. This allows for the testing of three hypotheses on the domain specificity of learning resources and their effect on learning activities and learning outcomes:

• Hypothesis 1 (mean differences): Educational and learning capitals for school learning and learning to play a musical instrument should differ in their availability.
• Hypothesis 2 (factor structure): In a confirmatory factor analysis, educational and learning capital related to learning in school and leaning a musical instrument can be identified as latent factors.
• Hypothesis 3 (correlations): Domain-congruent correlations should be higher than domain-incongruent correlations, i.e., educational and learning capital related to school learning and leaning a musical instrument should correlate more closely with their respective domain-related measures indicating successful learning for school or a musical instrument (i.e., grades, self-efficacy, practice time). However, no different correlation is expected with regard to the school grade in music, since educational and learning capital for school learning and educational and learning capital for learning a musical instrument should have a comparable effect.

## Materials and Methods

### Participants

A total of 365 students (222 girls and 143 boys; age: *M* = 13.1 years, *SD* = 2.27) from German schools volunteered to take part in the study. They all took musical instrument lessons organized by their school and were members of their school music orchestra. They had been playing their instrument for at least 2 years.

### Measures

#### Educational and Learning Capital: School

Educational and learning capital for the domain of school learning was measured with the Questionnaire of Educational and Learning Capital (QELC; see Vladut et al., 2013). Various studies prove its excellent psychometric properties (Paz-Baruch, 2015; Vladut et al., 2015; Arilena and Leana-Tacilar, 2016). The QELC measures each of the 10 capitals with the help of five items. The items were answered on a 6-point Likert-type scales ranging from 1 (not at all true) to 6 (absolutely true). A sample item for the organismic learning capital subscale reads “Being physically fit also helps me to learn and study for school for long periods of time.” A sample item from the economical educational capital subscale reads “My family spends more money on my schooling than other families do.” All ten subscales had an acceptable reliability with Cronbach’s alphas of at least 0.64.

#### Educational and Learning Capital: Music

To measure educational and learning capital for the domain of learning a musical instrument, we used an adapted version of the QELC (Ziegler et al., 2014) in which all items referred to learning a musical instrument (instead of learning for school). A sample item for the organismic learning capital subscale reads “Being physically fit also helps me to learn and study my musical instrument for long periods of time.” A sample item for the economical educational capital subscale reads “My family spends more money on my learning a music instrument than other families do.” The reliabilities of the reformulated scales were acceptable with Cronbach’s alphas of at least 0.68.

#### Academic Achievements

The students reported their grades on their last report card for the main subjects of mathematics, German language, and first foreign language (which are considered to be of special importance), as well as their grades in music. In German, the highest possible grade is 1 and the lowest possible grade is 6, with a grade of 5 or worse indicating failure to reach the classroom goal.

#### Self-Efficacy School and Self-Efficacy Music

Due to time constraints, it was only possible to measure self-efficacy with single items. Self-efficacy for school learning and self-efficacy for learning a musical instrument were measured on a 6-point Likert scale ranging from 1 (strongly disagree) to 6 (strongly agree). Sample items read: “If I want to, I can easily increase my school grades” and “If I want to, I can easily increase my music instrument performance.”

#### Practice of the Musical Instrument

Diary studies must be particularly economical, especially taking into account time constraints. For this reason, surveys are typically limited to a few minutes (Reis and Gable, 2000; Bolger et al., 2003). According to Reis and Gable (2000), daily entries should not exceed 5–7 min. For this reason, single item measures are often preferred (van Hooff et al., 2007). Practice of the musical instrument was measured in line with the sociotope approach (Ziegler et al., 2017b). For 7 days students filled out a learning diary. They answered for every waking hour (except for school hours) what activity they had carried out (including practicing their musical instrument in minutes). For each activity (i.e., for each time slot of an hour), students filled out two single items that referred to their normative action space (“Have you been expected to practice your instrument?”) and to their objective action space (“Would it generally have been possible for you to practice your musical instrument?”) concerning practicing their musical instrument. Answers were given on a 10-point scale from 1 (absolutely not) to 10 (absolutely).

### Data Collection

The QELC was administered at the beginning of the school year. School grades and self-efficacy were also measured at this time. The participants filled out the journal 6 weeks later. The reason for this time-delayed assessment was, first, that we wanted to rule out interferences between answering the QELC and the sociotope measures. Second, music-instrument lessons have been organized by the schools and restarted after summer holidays with the new school year. We assumed that after 6 weeks routines had been established.

### Data Analysis

To examine our assumptions about domain specificity of educational and learning capitals we conducted a Confirmatory Factor Analysis (CFA) with the twenty capital subscales. We built four latent factors for the school learning and music versions of the educational capitals and the learning capitals. For the combined capitals we expected co-variances.

We used the software R 3.5.0 with the library lavaan 0.6-1 (Rosseel, 2012; Rosseel et al., 2018). The lavaan library offers several methods to fit a latent or manifest variable model. The CFA was estimated with Full-Information-Maximum Likelihood (FIML). To examine goodness of fit of the model Chi-square Fit Statistics, Root Mean Square Error of Approximation (RMSEA), Comparative Fit Index (CFI) and Tucker-Lewis Index (TLI) were used.

For validation purposes, we calculated simple correlations between the four capital scales and academic achievements, self-efficacy, and practice of the musical instrument.

## Results

### Descriptive Statistics and *t*-Tests

Table 2 shows means, standard deviations and Cronbach’s alphas of the capital scales in the school learning and musical instrument learning version. Our first hypothesis was that there will be differences in the availability of the capitals in the two domains. Table 2 shows paired *t*-test results including Cohen’s d. With the exception of cultural educational capital, students indicated that they had more educational capital for learning their musical instrument than for school learning. 2-tailed paired samples *t*-tests showed that the mean differences are statistically significant, economic educational capital, *t*(364) = 6.54, *p* < 0.001; didactic educational capital, *t*(364) = 19.35, *p* < 0.001; social educational capital, *t*(364) = 8.37, *p* < 0.001; infrastructural educational capital, *t*(364) = 10.22, *p* < 0.001; cultural educational capital, *t*(364) = −2.48, *p* < 0.05. A very similar picture was found with learning capital. For all forms of learning capital, students indicated that they had more resources for learning the musical instrument than for learning for school, organismic learning capital, *t*(364) = 14.28, *p* < 0.001; actional learning capital, *t*(364) = 8.26, *p* < 0.001; telic learning capital, *t*(364) = 7.31, *p* < 0.001; episodic learning capital, *t*(364) = 5.65, *p* < 0.001; attentional learning capital, *t*(364) = 8.05, *p* < 0.001. However, after a control of Type I error by a Bonferroni adjustment, the mean difference in cultural educational capital reported by the students was no longer significant, *p* > 0.1.

**TABLE 2:** Descriptive statistics, Cronbach’s alpha of Educational Capital (EC) and Learning Capital (LC) scales, and paired t-test results.

Type of Scale	School version			Music version			*Paired t-test*	
Scales	*M*	*SD*	*Cronbach’s alpha*	*M*	*SD*	*Cronbach’s alpha*	*t(364)*	Cohen’s *d*
Economic EC	4.42	0.95	0.73	4.70	0.95	0.80	6.54**	0.30
Didactic EC	3.86	0.95	0.77	4.89	0.79	0.75	19.35**	1.18
Social EC	4.09	0.85	0.67	4.45	0.84	0.68	8.37**	0.43
Infrastructural EC	4.20	0.81	0.74	4.62	0.79	0.74	10.22**	0.54
Cultural EC	4.30	0.84	0.64	4.18	0.94	0.75	−2.48*	0.13
Educational Capital	4.18	0.69	0.84	4.56	0.72	0.89	11.76**	0.55
Organismic LC	3.72	1.03	0.76	4.42	0.92	0.80	14.28**	0.74
Actional LC	4.19	0.84	0.73	4.55	0.81	0.79	8.26**	0.44
Telic LC	3.68	0.94	0.68	4.05	0.96	0.71	7.31**	0.38
Episodic LC	4.10	0.88	0.79	4.37	0.89	0.83	5.65**	0.30
Attentional LC	3.55	0.94	0.79	3.97	1.04	0.83	8.05**	0.43
Learning Capital	3.85	0.77	0.89	4.27	0.80	0.92	11.07**	0.54

*, t-test is significant at the 0.05 level (2-tailed); **, t-test is significant at the 0.01 level (2-tailed).

Overall, the results of the *t*-tests clearly support our first hypothesis. Students possess different amounts of educational and learning capital in the two domains investigated.

### Confirmatory Factor Analysis

In hypothesis 2 we assumed that in a confirmatory factor analysis the two domains of musical instrument learning and school learning can be distinguished. This expectation was confirmed for both educational and learning capital. However, in line with previous studies (Vladut et al., 2013, 2015) and theoretical considerations (Ziegler and Baker, 2013), we found it plausible that some types of capital correlated with each other because they draw on the same learning resources.

**FIGURE 1:**
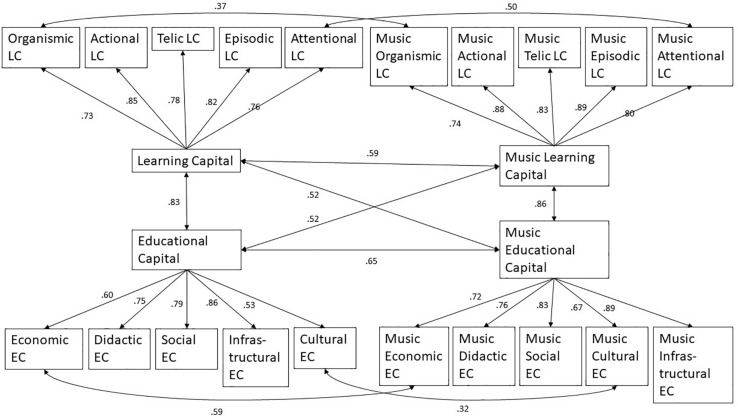
Confirmatory factor analysis.

The model with the best model fit is shown in Figure 1 and Table 3, which overall supports Hypothesis 2. To judge the fit of the model, the significant χ^2^ can be ignored, because with 365 cases, we have a much higher number than the limitation of 200 cases, allowing to use the χ^2^-test (Awang, 2015). The CFI in the range of 0.90 to 0.95 is acceptable (Brown, 2015) and the TLI close to 0.90 can be accepted if other fit indices are satisfactory. As the RMSEA is not above 0.10 and the SRMR is below 0.08, the model does not have to be rejected. Furthermore, the χ^2^/df ratio is below 5.0 (Wheaton et al., 1977).

**TABLE 3:** Results of the CFA.

χ^2^	df	χ^2^/df	*P* value (chi-square)	CFI	TLI	RMSEA	SRMR
658.898	159	4.144	0.000	0.909	0.891	0.093	0.054

The model is consistent with the assumption that educational and learning capitals are domain-specific. Within both domains, the individual educational capitals form a latent factor which is to be regarded as general educational capital of the respective domain. The same applies to the learning capital. These form a latent factor in their respective domain, too, which can be regarded as general learning capital in a domain. The individual educational capitals of a domain load only on the latent factor of their domain and neither on the latent factor learning capital of the same domain nor on the latent factor of the other domain. This also applies to the individual learning capitals of both domains with regard to the latent educational capital factors.

With regard to the individual educational and learning capitals, however, there are some co-variances across the domain boundaries, but only for the same type of capital. This means that individual capitals, such as the economic educational capital for school learning and the economic educational capital for learning a musical instrument have an undirected relationship. This is also true for cultural educational capital, attentional learning capital, and organismic learning capital.

### Correlations

In hypothesis 3, we assumed that educational and learning capital for school learning and learning to play a musical instrument correlate more closely with variables indicative of learning and learning outcomes in the respective domain. The correlations are shown in Table 4. To test whether two correlation coefficients differ significantly, Meng et al.’s z (1992) was used. Since we tested directed hypotheses, one-tailed testing was conducted.

**TABLE 4:** Correlations between domain-specific versions of the QELC and indicators of learning in school and of the musical instrument.

	Educational Capital	Learning Capital	Music Educational Capital	Music Learning Capital
School grade in math	−0.138*	−0.245**	–0.047	–0.105
School grade in German language	−0.154**	−0.240**	–0.013	–0.030
School grade in first foreign language	−0.231**	−0.297**	–0.085	−0.124*
School grade in music	–0.097	−0.130*	–0.068	–0.103
Self-efficacy school learning	0.313**	0.425**	0.193**	0.295**
Self-efficacy musical instrument learning	0.332**	0.357**	0.459**	0.547**
Objective action space	0.110	0.024	0.301**	0.209*
Normative action space	0.081	0.056	0.212*	0.270*
Practicing time	0.214	0.151	0.228*	0.310**

*, Correlation is significant at the 0.05 level (2-tailed); **, Correlation is significant at the 0.01 level (2-tailed).

With regard to school achievement, as expected, educational and learning capital for school learning correlated more closely with grades in mathematics, German language, and first foreign language than educational and learning capital for learning a musical instrument (educational capital for school learning vs. educational capital for learning a musical instrument: Math, *z* = 1.93, *p* < 0.05; German language, *z* = 2.99, *p* < 0.01; first foreign language, *z* = 3.13, *p* < 0.01; learning capital for school learning vs. learning capital for learning a musical instrument: Math, *z* = 2.91, *p* < 0.05; German language, *z* = 4.33, *p* < 0.01; first foreign language, *z* = 3.63, *p* < 0.01). Also as expected, the respective correlation coefficients did not differ significantly with regard to the grade in music; educational capital for school learning vs. educational capital for learning a musical instrument, *z* = −0.61, *p* > 0.1; learning capital for school learning vs. learning capital for learning a musical instrument, *z* = −0.55, *p* > 0.1.

The correlation pattern between the domain-specific versions of the QELC and self-efficacy of school learning and learning to play a musical instrument were also as expected. Availability of educational and learning capital for school learning was more strongly related to self-efficacy of school learning than to self-efficacy of learning a musical instrument, *z* = 2.64, *p* < 0.01, and *z* = −2.90, *p* < 0.01; while the availability of educational and learning capital for learning a musical instrument was more strongly related to self-efficacy of learning to play a musical instrument than to self-efficacy to learn for school, *z* = −2.99, *p* < 0.01 and *z* = −4.45, *p* < 0.01.

Finally, hypothesis 3 was also tested for practice of the musical instrument. In the learning diaries the students reported three aspects of their sociotopes with regard to learning the musical instrument: Objective action space, normative action space, and practice time. As these were assessed related to music, educational and learning capital for learning a musical instrument should correlate more strongly with them than educational and learning capital for school learning. The hypothesis was supported albeit with one exception, and with significant results in the expected direction for educational capital school vs. educational capital musical instrument: Objective action space, *z* = −4.14, *p* < 0.01; normative action space, *z* = −2.80, *p* < 0.01; practicing time, *z* = −0.31, *p* > 0.1; Learning capital school vs. learning capital musical instrument: Objective action space, *z* = −3.82, *p* < 0.01; normative action space, *z* = −4.48, *p* < 0.01; practicing time, *z* = −3.39, *p* < 0.01.

In summary, it can be noted that 17 out of the 18 comparisons of correlations were in the expected direction, including the correlations between the domain-specific availability of educational and learning capitals with the grade in music, where no differences were expected. After Bonferroni adjustment, 15 out of the 16 expected correlational differences were still significant. We regard this as a confirmation of Hypothesis 3, which implies that a domain-specific assessment of educational and learning capital might result in improved predictions in future studies.

## Discussion

This work started from two theoretical premises. The first premise was that talent development is highly dependent on the availability of learning resources. On the one hand, this had been derived from the observation that clusters are observed on many levels of analysis (Ziegler and Baker, 2013). On the other hand, it was based on research studies which demonstrated the role of learning resources for talent development in general, and educational and learning capital in particular (Vladut et al., 2013, 2015; Paz-Baruch, 2015, 2020; Phillipson et al., 2017; Stoeger et al., 2017b; Vialle, 2017; Lafferty et al., 2020).

The second premise of this work was that there are specific ensembles of potent resources for specific learning goals and thus talent domains. Therefore, though there might be a substantial overlap of the resources needed for successful learning in one domain, these might not be identical with the resources needed to be successful in another domain. For example, the resources that lead to a successful learning career in school might not be identical to the resources needed for a successful learning career in music. This insight had been already implicitly taken into account within the ELCA. For example, if resources were investigated in a certain domain, the QELC was always adapted to the specific domain (Ziegler et al., 2014, 2019). What was missing, however, was a study that shows the different benefits of educational and learning capital for different domains.

Therefore, the goal of our study was to investigate the domain specificity of the ELCA. We decided to use a within-subject design. This allows more convincing demonstration that individuals use learning resources specifically for certain domains. However, this raises the problem of choosing appropriate domains. For reasons of expediency, we chose a domain in which everyone in our country participates, school-based learning in secondary education, and a domain in which many participate, learning a musical instrument. Two aspects are important in this decision to assess the relevance of the study.

First, the participants in our study were far from a degree of talent development that represents eminence. In terms of learning at school they had, on average completed only just over half of their schooling. Before eminence can be reached, or the extremely long periods of deliberate practice required can be achieved, many more years of engagement are necessary (Ericsson and Harwell, 2019). Similarly, the study participants were only at the beginning of the musical instrument lessons. Although they had been learning the instrument for at least 2 years, only very few children had had instrumental lessons for more than 4 years. This is also far from the time of practice considered necessary before eminence can be achieved (Ericsson et al., 1993). It can be assumed that the further that learning in a domain is from eminence, the less specialized it is (Debatin et al., 2015). If the need to take the domain into account can be shown at what is actually a fairly early stage of talent development, then this a fortiori applies to all later stages of talent development, which presumably require higher levels of specialization.

The second important reason for choosing the two domains was that although they are sufficiently different, they also share commonalities in terms of learning resources. The school organized the instrumental lessons, which in some cases meant that the school music teacher was also the music instrument teacher. The school’s offer to learn a musical instrument was aimed primarily at students who were able to cope well with the school requirements, who had parental support in both domains and who were motivated for both domains themselves. The musical instrument lessons were designed to be compatible with the school in several ways, including the time of the musical instrument lessons, which took place in the school building. Finally, there was overlapping of content such as the ability to read notes. Thus, if even for domains with obvious overlapping of learning resources the need to take their specificity into account can be shown, then this applies a fortiori to other domains with less overlap.

Three hypotheses were tested in the study. The first hypothesis postulated that the learning resources for school learning and learning of a musical instrument differ in terms of availability. This hypothesis could be supported by simple mean value comparisons of the five forms of learning capital and the five forms of educational capital for the two domains. Although it was not an explicit hypothesis of our study, it is worth noting that nine of the ten mean comparisons indicated that students had more learning resources with regard to learning the musical instrument. This makes perfect sense, because accepting an additional offer from the school is particularly beneficial, if one expects successful participation.

The second hypothesis postulated the factorial validity of educational capital and learning capital in the domains of school learning and learning to play a musical instrument. To this end, a confirmatory factor analysis was conducted. The confirmatory factor analysis showed that educational and learning capitals for school learning and for the learning of a musical instrument are different factors. As expected, the educational and learning capitals related to school learning and to learning a musical instrument each form a latent factor. Some plausible co-variances were found in individual education and learning capitals across the domain boundaries. However, this concerned the same type of capital in each case. Thus, economic educational capital, cultural educational capital, organismic learning capital, and attentional learning capital for school learning and learning to play the musical instrument may overlap. For example, some free hours in the afternoon are basically available for both academic learning and practicing the musical instrument.

In hypothesis 3, domain-congruent correlations were postulated between the capitals and various indicators of school learning and the learning of a musical instrument. As expected, educational and learning capital for school learning was significantly higher correlated with better grades in mathematics, in the German language and in the first foreign language than educational and learning capital for learning a musical instrument. The correlations of learning resources in both domains with music grades did not differ significantly from each other. This seems plausible, as grades in music seem to have a special status and resources from both domains might be useful for reaching good grades in the subject of music.

Domain-congruent correlations were also found for the capitals with regard to the self-efficacy of school learning and learning to play the musical instrument. As expected, educational and learning capital for school learning better predicted the self-efficacy for school learning and educational and learning capital for learning the musical instrument better predicted the self-efficacy for learning a musical instrument.

Hypothesis 3 also addressed several variables important from the perspective of the sociotope approach (Ziegler et al., 2017b): time spent practicing the musical instrument, time spent in situations where the students could potentially practice their instrument (objective action space), and times students perceived to be expected to or important for them to practice their instrument (normative action space). As expected the educational and learning capital for learning a musical instrument correlated more strongly with these variables than educational and learning capital for school learning, with one exception. Educational and learning capital for learning a musical instrument did not significantly correlate with the objective action space for practicing a musical instrument. A possible explanation for this unexpected finding might be an exceeded threshold value with regard to educational capital, i.e., exogenous learning resources. It seems feasible that parents only make the decision to allow their child to attend voluntary musical instrument lessons if exogenous learning resources are available in sufficient quantity. However, though the availability of exogenous learning resources might provide a sufficient objective action space for practicing a musical instrument, this does not automatically mean that it goes along with a normative expectation to use this opportunity (normative action space).

All in all, our study contains numerous findings that broaden the research on learning and educational capital and that support the domain-specificity of educational and learning capital with regard to talent development. However, our study also has various limitations.

A first limitation of our study lies in relying on self-reports from questionnaires and diaries. A more objective recording of resources would be definitely desirable. Also, some aspects in our study, especially in the diary study, were measured with single items. Here, too, a replication of our study with more reliable measuring instruments would be desirable.

A second limitation are the fit indices of the confirmatory factor analysis. Although they were still satisfactory, they were certainly not perfect. Therefore, a replication of the findings of our study would be desirable.

A third limitation of our study is the partial use of single items. However, it distinguishes between the learning in the two domains.

From a theoretical standpoint, a fourth limitation of our study lies in the fact that the domain specificity of learning resources was only shown for two domains, and at a rather early stage of talent development. To ensure the generalizability of our finding to other domains and other stages of talent development further studies are needed.

A final limitation lies in the fact that the design of our study does not allow conclusions to be drawn about the direction of influence between the variables under investigation. Although the recording of educational and learning capital was carried out weeks before the diary study, this does not indicate causality in the sense of educational and learning capital influencing the shape of dependent variables in the statistical analyses. Indeed, ELCA is committed to the concept of circular causality, which rejects such simple cause-effect relations (Bateson, 1972; Ziegler and Stoeger, 2017a) that, however, with a design like ours could not be investigated.

## Data Availability Statement

The datasets presented in this article are not readily available because of local data protection regulations. Requests to access the datasets should be directed to Marold.Reutlinger@fau.de.

## Ethics Statement

Ethical review and approval was not required for the study on human participants in accordance with the local legislation and institutional requirements. Written informed consent from the participants’ legal guardian/next of kin was not required to participate in this study in accordance with the national legislation and the institutional requirements.

## Author Contributions

All authors listed have made a substantial, direct and intellectual contribution to the work, and approved it for publication.

## Conflict of Interest

The authors declare that the research was conducted in the absence of any commercial or financial relationships that could be construed as a potential conflict of interest.

## Footnotes

1 The term “golden ages” refers to periods of highest development of a culture or a heyday of a certain form of cultural creation.
